# Remodeling of calcium signaling in tumor progression

**DOI:** 10.1186/1423-0127-20-23

**Published:** 2013-04-17

**Authors:** Yih-Fung Chen, Ying-Ting Chen, Wen-Tai Chiu, Meng-Ru Shen

**Affiliations:** 1 Department of Pharmacology, National Cheng Kung University, Tainan, Taiwan; 2 Department of Biomedical Engineering, National Cheng Kung University, Tainan, Taiwan; 3 Institute of Basic Medical Sciences, National Cheng Kung University, Tainan, Taiwan; 4 Advanced Optoelectronic Technology Center, National Cheng Kung University, Tainan, Taiwan; 5 Infectious Diseases and Signaling Research Center, National Cheng Kung University, Tainan, Taiwan; 6 Department of Obstetrics & Gynecology, National Cheng Kung University Hospital, Tainan, 704, Taiwan

**Keywords:** Migration, Ca^2+^ homeostasis, Stromal interaction molecule (STIM), Orai, Transient receptor potential (TRP) channels

## Abstract

Intracellular Ca^2+^ is one of the crucial signalings that modulate various cellular functions. The dysregulation of Ca^2+^ homeostasis has been suggested as an important event in driving the expression of the malignant phenotypes, such as proliferation, migration, invasion, and metastasis. Cell migration is an early prerequisite for tumor metastasis that has a significant impact on patient prognosis. During cell migration, the exquisite spatial and temporal organization of intracellular Ca^2+^ provides a rapid and robust way for the selective activation of signaling components that play a central role in cytoskeletal reorganization, traction force generation, and focal adhesion dynamics. A number of known molecular components involved in Ca^2+^ influx pathways, including stromal interaction molecule (STIM)/Orai-mediated store-operated Ca^2+^ entry (SOCE) and the Ca^2+^-permeable transient receptor potential (TRP) channels, have been implicated in cancer cell migration and tumor metastasis. The clinical significance of these molecules, such as STIM proteins and the TRPM7 channel, in tumor progression and their diagnostic and prognostic potentials have also been demonstrated in specific cancer types. In this review, we summarize the recent advances in understanding the important roles and regulatory mechanisms of these Ca^2+^ influx pathways on malignant behaviors of tumor cells. The clinical implications in facilitating current diagnostic and therapeutic procedures are also discussed.

## Review

The ubiquitous second messenger Ca^2+^ is an important signaling for several fundamental physiological functions, such as cell cycle control, survival, apoptosis, migration, and gene expressions [1]. Regulation of intracellular Ca^2+^ ([Ca^2+^]_i_) homeostasis involves both Ca^2+^ entry from the extracellular space and Ca^2+^ release from the intracellular stores, such as the endoplasmic reticulum (ER) or mitochondria [2]. Some human diseases have been linked with the abnormal regulation of Ca^2+^ homeostasis, including developmental disorders, hypertension, cardiovascular disease, diabetes, Alzheimer's disease, and cancer [3,4]. Although changes in Ca^2+^ homeostasis may not be a necessity for malignant initiation, the altered Ca^2+^ signalings in cancer cells contribute to important events during tumor progression, such as proliferation, migration, invasion, and metastasis [5,6]. Understanding the remodeling of Ca^2+^ homeostasis in cancer cells may thus shed a light on the potential therapeutic targets or prognostic biomarkers. This review focuses on the important roles and regulatory mechanisms of Ca^2+^ influx pathways in aggravating tumor malignant behaviors, particularly in cellular migration and tumor metastasis.

### Cell migration: a dynamic process between focal adhesion turnover, cytoskeletal dynamics and cell contractility

Cell migration and invasion are a prerequisite for tumor metastasis which has a great impact on cancer patient outcomes. The migratory ability assists cancer cells in escaping from their primary sites of origin, and contributes to their dissemination through nearby circulations. The spatial and temporal coordination of cell-substrate adhesion, actin cytoskeleton, non-muscle myosin II-mediated contraction and cell-substrate detachment are required for cell migration [7,8]. Central to this process is the structural and signaling linkage complexes between the extracellular matrix (ECM) and cytoskeleton that are known as focal adhesions [9,10]. The speed of the coordinated and dynamic formation and disassembly of focal adhesions determines the efficiency of cell migration. The association between actin filaments and myosin II forms contractile actomyosin fibers [11]. Migratory cells generate intracellular forces to support the rear-end retraction and forward protrusion through the myosin II-based actomyosin contractility. The transmission of myosin II-based actomyosin contraction to focal adhesions establishes the contractile force that relocates the cell body and contributes to cell locomotion [12]. These contractile forces are important for the regulation of focal adhesion turnover and cytoskeletal organization, and thus mediate the efficient cell migration.

### Ca^2+^ signaling in cell migration

In addition to the extracellular chemoattractant stimulations, cell migration depends on the spatially and temporally coordinated intracellular Ca^2+^ signaling [13]. During cell migration, Ca^2+^ signaling has a multifunctional role in directional sensing, cytoskeleton redistribution, traction force generation, and relocation of focal adhesions [14]. Polarized, migrating cells exhibit a rear-to-front gradient of [Ca^2+^]_i_, with higher concentration at the rear end of a migrating cell, which is thought to be responsible for rear-end retraction [15]. On the other hand, spatially and temporally confined repetitive changes in [Ca^2+^]_i_, termed as Ca^2+^ flickers or Ca^2+^ microdomains, are enriched near the leading edge of migrating cells and implicated in controlling cycles of lamellipodia retraction and strengthening local adhesion to ECM [16-18]. The rear-end retraction is supported by the processes of actomyosin contraction and focal adhesion disassembly that are regulated by Ca^2+^-dependent signalings. The myosin II-based actomyosin contraction is mainly mediated by myosin light-chain (MLC) phosphorylation through the Ca^2+^-dependent MLC kinase (MLCK) [19]. The disassembly of cell adhesions is due to the cleavage of focal adhesion proteins, such as integrins, talin, vinculin and focal adhesion kinase (FAK), by the Ca^2+^-dependent protease, calpain [20,21]. Additional biochemical links between [Ca^2+^]_i_ and the focal adhesions is provided by the focal adhesion-localized proline-rich tyrosine kinase 2 (Pyk2), which requires Ca^2+^ for activation [22]. The Ca^2+^-dependent activation of calpain and Pyk2 thus regulates multiple signaling events crucial for the focal adhesion turnover and cell migration.

### Ca^2+^ influx pathways in cell migration and tumor metastasis

Transient changes in [Ca^2+^]_i_ play an important role in various cellular processes associated with cancer cell migration and tumor metastasis [14]. The spatially-confined sustained or transient increases of Ca^2+^ concentration can occur in the form of waves, spikes or oscillations [23]. Such increases occur as a result of Ca^2+^ entry through plasma membrane Ca^2+^-permeable channels or Ca^2+^ mobilization from internal stores, such as the ER, through the ryanodine receptor (RYR) and/or the inositol-1,4,5-trisphosphate receptor (IP3R) channels [24]. Several plasmalemmal Ca^2+^ channels have been suggested to play important roles in regulating cancer cell migration and tumor metastasis, as summarized in Table 1. The emerging role of dysregulated mitochondrial Ca^2+^ homeostasis in tumorigenesis has also received increasing attention [25-28]. The reduction of mitochondrial Ca^2+^ uptake in cancer cells can decrease the activation of the mitochondrial intrinsic apoptosis pathway, and thus favors cancer cell survival and tumor metastasis. This review focuses on the important role of stromal interaction molecule (STIM)/Orai-mediated store-operated Ca^2+^ entry (SOCE) and the Ca^2+^-permeable transient receptor potential (TRP) channels on tumor malignant behaviors.

**Table 1:** **Role of plasmalemmal Ca**^**2+ **^**channels in cell migration and tumor metastasis**

**Ca**^**2+**^**channel**	**Cell type**	**Mechanisms and effectors**	**References**
***Store-dependent SOC channels***			
STIM1-Orai1	• Human cervical cancer SiHa and CaSki cells	• Increase in EGF-stimulated cellular migration and invasion	[29,30]
		• Increase in focal adhesion dynamics through the Ca^2+^-regulated protease calpain and cytoplasmic kinase Pyk2	
		• Upregulation of EGF-induced MLC phosphorylation and actomyosin reorganization	
		• Upregulation of VEGF production	
		• Promotion of tumor growth and angiogenesis in a xenograft mice model	
	• Human breast cancer MDA-MB-231 cells and mouse mammary tumor 4 T1 cells	• Increase in serum-induced cellular migration and invasion	[31]
		• Increase in focal adhesion turnover rates through Ras and Rac1	
		• Promotion of tumor growth and metastasis in a xenograft mice model	
STIM-Orai3	• Human breast cancer MCF7 cells (ER^+^ breast cancer cells)	• Increase in anchorage-independent growth and Matrigel invasion	[32,33]
		• Increase in tumorigenesis in a xenograft mice model	
***Store-independent SOC channel***			
SPCA2-Orai1	• Human breast cancer MCF-7 cells	• Constitutively active store-independent Ca^2+^ influx	[34]
		• Promotion of proliferation and colony formation	
		• Increase in tumorigenesis in a xenograft mice model	
***TRP channels***			
TRPM1	• Murine melanoma B16-F1 cells	• Reduce in tumor metastasis	[35,36]
TRPM7	• Human breast cancer MDA-MB-231 cells and MEF cells	• Increase in cellular migration	[18,37-39]
		• Guidance of polarized cellular migration	
		• Increase in peripheral focal adhesion turnovers through the Ca^2+^-regulated protease m-calpain	
		• Inhibition of myosin II-based cell contractility	
		• Increase in tumorigenesis in a xenograft mice model	
	• Human nasopharyngeal cancer 5-8 F and 6-10B cells	• Increase in cellular migration	[40]
	• Human lung cancer A549 cells	• Increase in EGF-stimulated cellular migration	[38]
TRPM8	• Human prostate cancer PC-3 cells	• Decrease in cellular migration	[41,42]
		• Inactivation of FAK	
TRPV1	• Human hepatoblastoma HepG2 cells	• Increase in HGF-stimulated cellular migration	[43,44]
TRPV2	• Human prostate cancer LNCaP and PC-3 cells	• Increase in cellular migration and invasion	[45]
		• Induction of invasive enzymes MMP-2, MMP-9 and cathepsin B	
		• Increase in tumorigenesis in a xenograft mice model	
TRPC6	• Human glioblastoma cells	• Increase in cell proliferation through regulation of CDK1 activation and Cdc25C expression	[46,47]
		• Increase in anchorage-independent growth and Matrigel invasion	
		• Increase in endothelial cell tube formation	
		• Increase in tumorigenesis in a xenograft mice model	

Cdc25C, cell division cycle 25 homolog C; CDK1, cyclin-dependent kinase 1; EGF, epidermal growth factor; ER, estrogen receptor; FAK, focal adhesion kincase; HGF, hepatocyte growth factor; SPCA2, secretory pathway Ca^2+^-ATPase; MEF, mouse embryonic fibroblast; MMP, matrix metalloproteinase; Pyk2, proline-rich tyrosine kinase 2; VEGF, vascular endothelial cell growth factor.

#### STIM/Orai in SOCE activation and Ca^2+^ homeostasis

**Figure 1:**
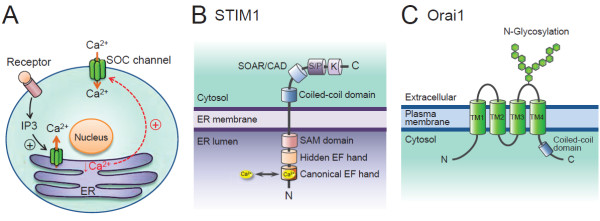
**Regulatory mechanism of [Ca**^**2+**^**]**_**i **_**homeostasis. (A)** Store-operated calcium entry (SOCE) in non-excitable cells. The activation of surface receptors stimulates phospholipase C (PLC) to increase the second messenger inositol-1,4,5-trisphosphate (IP3), which binds to the IP3 receptor in the endoplasmic reticulum (ER) membrane and causes rapid and transient Ca^2+^ release from the ER lumen. The decrease in ER luminal Ca^2+^ results in the opening of the plasmalemmal store-operated Ca^2+^ (SOC) channel, leading to the elevated intracellular Ca^2+^ levels ([Ca^2+^]_i_). **(B)** Domain architecture of the ER Ca^2+^ sensor stromal interaction molecule 1 (STIM1) protein. STIM1 is a single-transmembrane protein that is mainly localized in the endoplasmic reticulum (ER). The luminal N-terminus contains a canonical EF hand motif that binds Ca^2+^, a hidden EF hand that does not bind Ca^2+^, and a sterile α-motif (SAM) domain that is important for STIM1 oligomerization. The cytosolic C-terminus contains the coiled-coil domains, a STIM-Orai activating region (SOAR) or CRAC activation domain (CAD), and serine or proline (S/P)-rich segments and lysine (K)-rich clusters. The SOAR/CAD domain is essential for the gating of Orai1. The predicted protein-protein interaction domains in STIM1 include the SAM domain, coiled-coil domains, SOAR/CAD domain, S/P-rich segments and K-rich clusters. **(C)** Predicted topology of the plasmalemmal SOC channel Orai1. Orai1 consists of four transmembrane domains (TM1-TM4) and intracellular N- and C-termini. It is suggested that the TM1 lines the central pore of Orai1 channel. The short C-terminal putative coiled-coil domain is important for binding to the SOAR/CAD domain of STIM1, and its disruption impairs STIM1-mediated activation of Orai1 channel. Hexagonal structures represent glycosyl residues attached to an arginine (N)-linked glycosylation site (N^223^).

SOCE is a predominant pathway of Ca^2+^ entry in non-excitable cells, and is widely distributed in various cell types [48]. SOCE, by definition, is activated by Ca^2+^ release from the internal store. SOCE-mediated Ca^2+^ influx provides Ca^2+^ not only for ER store refilling, but also for signaling purposes. As shown in Figure 1A, the SOCE activation includes several steps: (1) Stimulation of G protein-coupled receptors or receptor protein tyrosine kinases activates phospholipase C, which hydrolyzes phosphatidylinositol bisphosphate to release the second messenger inositol-1,4,5-trisphosphate (IP3). (2) Binding of IP3 to IP3 receptors in the ER membrane causes a rapid and transient Ca^2+^ release from ER lumen. (3) The decrease in ER luminal Ca^2+^ activates store-operated Ca^2+^ (SOC) channels in the plasma membrane, leading to a sustained influx of extracellular Ca^2+^ across the plasma membrane [49].

**Figure 2:**
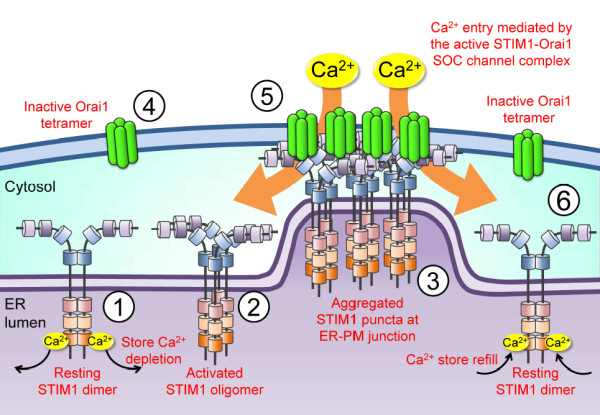
**STIM1-dependent SOCE activation.** STIM1 exists as the dimer maintained by the intermolecular interactions between its coiled-coil domains in the resting state **(①)**. Depletion of ER luminal Ca^2+^ causes Ca^2+^ dissociation from the STIM1 N-terminal canonical EF hand, leading to STIM1 oligomerization due to the intermolecular interactions between EF-SAM domains **(②)**. This activated STIM1 puncta interacts with the plasma membrane (PM) by the C-terminal polybasic K-rich clusters and accumulates in the ER-PM junctions **(③)**. Orai1 tetramers diffusing in the PM **(④)** are tethered and trapped in junctions by the electrostatic interaction between CAD/SOAR domain in STIM1 and basic domains in the C-terminus of Orai1. The formation of the active STIM1/Orai1 complex conformationally gates the opening of SOC channel Orai1, thereby allowing Ca^2+^ entry **(⑤)**. Upon store refilling, re-association of Ca^2+^ with STIM1 reverses EF-SAM oligomerization, causing STIM1-Orai1 uncoupling, Orai1 deactivation and the release of resting STIM1 dimers from puncta to redistribute throughout the ER **(⑥)**.

Two main molecular components have been identified as essential for SOCE activation [50-53]: STIM molecules as the ER Ca^2+^ sensors and Orai proteins as the pore-forming subunits of the plasmalemmal SOC channel (Figure 1B &1C). Mammals possess three types of Orai proteins (Orai1-3) [53,54], of which Orai1 is the most extensively characterized. Although all three Orai proteins form functional SOC channels when co-expressed with STIM1, they differ in tissue distribution and in the selectivity and conductivity for Ca^2+^[54]. STIM molecules, STIM1 and STIM2, are the single-transmembrane proteins that are mainly localized in the ER membrane. As shown in Figure 1B, the important functional domains of STIM1 include a canonical EF hand Ca^2+^-binding domain and a sterile α-motif (SAM) protein interaction domain in the luminal N-terminal end, and a STIM-Orai activating region (SOAR), which is similar to the CRAC activation domain (CAD), in the cytoplasmic C-terminal end [55]. The EF hand domain enables STIM1 to sense small decreases in ER luminal Ca^2+^ concentration, whereas the SAM domain mediates the STIM1 oligomerization. As depicted in Figure 2, once ER Ca^2+^ is depleted, STIM1 proteins oligomerize into multiple punctae and redistribute to the close proximity of plasma membranes, known as the ER-plasma membrane junctions. Orai1 protein, a four-transmembrane domain Ca^2+^ channel in the plasma membrane, translocates to the STIM1-containing ER-plasma membrane junctions following store depletion and opens to mediate Ca^2+^ entry [56]. The opening of the Orai1 Ca^2+^ channel is mediated by the direct physical interaction between the cytoplasmic C-terminal coiled-coil domain of Orai1 and the cytoplasmic C-terminal SOAR/CAD domain of STIM1. Although STIM2 molecule exhibits significant homology in the overall structure and basic properties with regard to STIM1, such as ER localization, luminal Ca^2+^ binding and redistribution to puncta at ER-plasma membrane junctions upon Ca^2+^ store depletion, its role in SOCE activation remains controversial [55]. It has been recently reported that different agonists activate different STIM proteins to sustain Ca^2+^ signals and downstream responses [57].

The physiological and pathological importance of SOCE has been implicated in many diseases, especially in immune disorders. SOCE is critical for the development and function of regulatory T cells and the formation of “immunological synapses” between T lymphocytes and antigen-presenting cells [58,59]. STIM1 or Orai1-deficiency causes several autoimmune diseases and myopathy in human subjects and mouse models [60,61]. Mast cells lacking either STIM1 or Orai1 exhibit defective cytokine production and release, which jeopardize allergic responses [62,63]. During phagocytosis, the recruitment of STIM1 towards phagosomes is required for the opening of phagosomal Ca^2+^ channels that generates localized Ca^2+^ elevations to promote high-efficiency phagocytosis [64]. In the lung, STIM1-Orai1 upregulation leads to an increase in pulmonary smooth muscle cell proliferation and in endothelial cell migration and vessel formation [65,66]. During lactation, the expression of Orai1 and STIM2 in mouse mammary glands is increased, whereas STIM1 is downregulated, indicating that Orai1-dependent SOCE may be one of the important Ca^2+^ influx routes to meet Ca^2+^ transport demand during lactation [67].

#### STIM1 controls cancer cell migration by regulating focal adhesion turnover and actomyosin contractility

**Figure 3:**
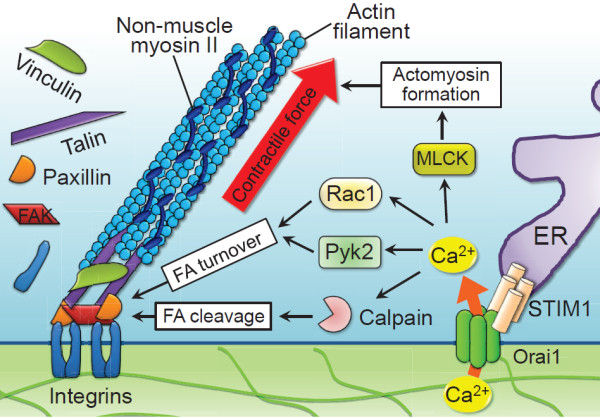
**STIM1-mediated Ca**^**2+ **^**influx regulates cell migration through focal adhesion turnover and actomyosin contractility.** The dynamic interactions among cytoskeleton, non-muscle myosin II and cell-substrate adhesion regulate cell migration. The interaction between ER Ca^2+^ sensor STIM1 and plasma membrane SOC channel Orai1 induces Ca^2+^ influx, which integrates the dynamic interactions between actomyosin and focal adhesion (FA) to mediate efficient cell migration. STIM1/Orai1-mediated Ca^2+^ influx regulates actomyosin formation through the Ca^2+^-dependent myosin light chain kinase (MLCK), accelerates FA turnover through the small GTPase Rac1 and the Ca^2+^-dependent proline-rich tyrosine kinase 2 (Pyk2), and promotes calpain-mediated disassembly and cleavage of FA proteins.

The molecular mechanisms of SOCE in regulating cancer cell migration have been emerged from studies on breast and cervical cancer cells [29,31]. Yang *et al.* provided evidence for the role of STIM1 and Orai1 in the migration of breast cancer cells [31]. Blocking SOCE, by a pharmacological inhibitor SKF96365 or by siRNA-mediated silencing of STIM1 or Orai1, impaired the focal adhesion turnover and invasive migrations of breast cancer cells. These defects of focal adhesion turnover and cell migration could be rescued by the constitutively active forms of the small GTPases Ras and Rac1 [29]. STIM1-dependent Ca^2+^ signalings also play an important role in epidermal growth factor (EGF)-stimulated cervical cancer cell migration [29]. EGF, an important stimulator for cancer cells migration [68], can stimulate the aggregation and translocation of STIM1 towards to the Orai1-containing regions of plasma membrane to mediate SOCE. STIM1-dependent SOCE is necessary for the activation of Ca^2+^-dependent protease calpain and tyrosine kinase Pyk2, which regulate the focal-adhesion dynamics of migratory cervical cancer cells. More importantly, STIM1-dependent Ca^2+^ signalings control cervical cancer cell migration by the regulation of actomyosin reorganization in conjunction with enhanced contractile forces [30]. STIM1 silencing inhibited the recruitment and association of active FAK and talin at focal adhesions, indicating the blockade of force transduction from integrin signaling. Furthermore, EGF-induced MLC phosphorylation and actomyosin reorganization were abolished by STIM1 knockdown and SOCE inhibitors. The direct measurement of cell traction forces revealed that STIM1-dependent Ca^2+^ signaling regulates the traction force generation at cell adhesions. The results from these studies suggest that STIM1-dependent Ca^2+^ signaling could integrate the dynamic interactions between actomyosin and focal adhesion to mediate efficient cell migration. The related mechanisms at least partly involve the modulation of focal adhesion turnover through the Ca^2+^-dependent Pyk2 and the small GTPase Rac1, focal adhesion protein cleavage through the Ca^2+^-dependent protease calpain, and actomyosin formation through MLC phosphorylation (as summarized in Figure 3). The significance of STIM1 in cellular migration may extend beyond breast and cervical cancer given its role in the migration and focal adhesion turnover in hepatocarcinoma cells [69]. Targeting the molecular components of SOCE, STIM1 and Orai1, is thus a promising approach to inhibit cancer cell migration and tumor metastasis.

#### Tumor STIM1 levels enhance metastatic potentials

**Figure 4:**
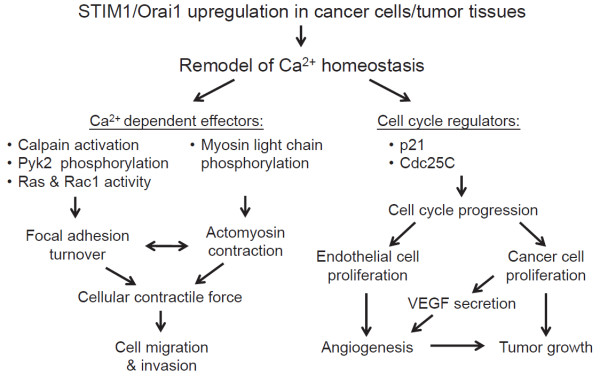
**STIM1/Orai1-mediated Ca**^**2+ **^**signalings in tumor biology.** Ca^2+^ homeostasis is remodeled in cancer cells during tumor progression, with Ca^2+^ influx increasing through STIM1/Orai1upregulation. STIM1/Orai1-dependent Ca^2+^ signaling integrates the dynamic interactions between actomyosin contraction and focal adhesion turnover to mediate efficient cell migration. STIM1 also influences cancer cell proliferation through cell cycle regulators p21 and cdc25C. Additionally, STIM1-mediated SOCE plays an important role in tumor angiogenesis; one possible mechanism involves the production of vascular endothelial growth factor (VEGF) from cancer cells, another is the cell cycle progression of vascular endothelial cells [72]. Accordingly, STIM1/Orai1-remodeled Ca^2+^ homeostasis is important for aggravating tumor development *in vivo.*

The functional significance of STIM1 and Orai1 in tumor progression *in vivo* has been revealed in breast and cervical cancer [29,31]. Consistent with the pro-migratory role of STIM1 and Orai1, suppressed expression levels of STIM1 and Orai1 in highly metastatic breast cancer cells inhibited lung metastasis after tail vein injection in immunodeficient SCID mice, which can be mimicked by pharmacological inhibitor SKF96365 [31]. Moreover, STIM1-dependent Ca^2+^ signaling plays an important role in tumor growth and angiogenesis *in vivo*[29]. Angiogenesis, the recruitment and formation of new blood vessels, is an essential step for tumor metastasis [70]. Through these vessels, tumor cells can exit the primary sites of origin, enter into the systemic circulation, and disseminate to distant organs. STIM1 overexpression significantly enhanced tumor growth, local spread and angiogenesis of human cervical cancer xenograft in SCID mice, whereas shRNA-mediated knockdown of STIM1 significantly decreased tumor growth and tumor vessel numbers [29]. It is thus proposed that tumor STIM1 overexpression may benefit the locomotion and metastasis of cancer cells. Consistently, the intraperitoneal administration of SOCE inhibitors, such as 2-APB and SKF96365, into human cervical cancer-bearing mice could cause tumor growth regression accompanied by the obliteration of tumor feeding vessels [29], suggesting both tumor cells and tumor vessels are the possible targets by SOCE inhibition. Mechanistic investigations revealed that the secretion of vascular endothelial growth factor (VEGF), a critical stimulator for tumor angiogenesis [71], from cervical cancer cells was dependent on their STIM1 expressions [29]. The results from these studies demonstrate the crucial role of STIM1-mediated Ca^2+^ influx in aggravating tumor development *in vivo* (Figure 4), especially in tumor angiogenesis and metastasis. Blocking Orai1- and STIM1-dependnet Ca^2+^ signaling is thus a potential strategy for cancer therapy.

#### Tumor STIM1 protein level has diagnostic and prognostic value

The clinical significance of SOCE in tumor progression has been demonstrated in cervical cancer [29]. Among 71% cases of early-stage cervical cancer examined, STIM1 protein expression was upregulated in cervical cancer tissues but rarely detected in adjacently non-cancerous cervical epithelia. The levels of STIM1 expression in tumor tissues were closely correlated with the primary tumor size, an important indicator of human cervical carcinoma progression *in vivo*[73]. In addition, cervical cancer patients with pelvic lymph-node metastasis, which is the primary cause of treatment failure and subsequent death in cervical cancer patients, displayed higher STIM1 expression in tumor tissues. More importantly, a significantly poorer 5-year overall survival rate was also found in primary tumors with STIM1 upregulation. Another study, based on the microarray data analyses of 295 breast cancer patients, also showed that transcriptionally-defined basal-like tumors, which have a poor prognosis and lack of effective therapies [74], are characterized by high STIM1 and low STIM2 mRNA expressions [67]. An increase in STIM1/STIM2 gene expression ratio has been associated with reduced survival in breast cancer patients. However, this trend of redundancy between STIM1 and STIM2 expressions was not noted in cervical cancer patients [29], indicating the role of STIM2 in tumor biology may be tissue- or cell-type specific. Taken together, these studies suggest a potential role for STIM1 protein as a diagnostic biomarker to predict the occurrence, progression or prognosis of cancer patients.

#### SPCA2/Orai1-mediated store-independent Ca^2+^ influx in breast tumorigenesis

In addition to STIM1-mediated activation of Orai1, an entirely different signalling mechanism, in which the Ca^2+^ influx through Orai1 is independent of store depletion, has recently been implicated in human breast tumorigenesis (Table 1) [34]. This mechanism involves the interaction of Orai1 with the Golgi-localized secretory pathway Ca^2+^ ATPase isoform 2 (SPCA2). SPCAs, including the ubiquitously expressed SPCA1 isoform and more restricted SPCA2 isoform [75,76], are ATP-powered Ca^2+^ pumps that transport Ca^2+^ into the Golgi luman for protein sorting and processing [77]. The limited distribution of SPCA2 includes the lumenal secretory cells of the mammary gland, where it normally functions in the regulation of Golgi Ca^2+^ levels and is drastically upregulated during lactation [78]. However, the contribution of SPCA2 to breast tumor progression is not through its conventional role in Golgi Ca^2+^ sequestration. Consistent with the overexpression of Orai1 in breast cancer [67], SPCA2 was overexpressed in human breast cancer cell lines and human breast tumors, whereas SPCA1 levels were similar among all cell lines examined [34]. Additionally, SPCA2 silencing attenuated basal [Ca^2+^]_i_, cell proliferation, anchorage-independent growth and mammary tumor formation in nude mice, whereas SPCA2 overexpression increased basal [Ca^2+^]_i_ and promoted breast tumorigenicity. Surprisingly, SPCA2 induced Ca^2+^ influx independently of its ATPase function, as a SPCA2 mutant with impaired Ca^2+^-ATPase activity increased basal [Ca^2+^]_i_ and anchorage-independent growth to a similar extent to that of wild-type SPCA2. The results from immunofluorescent staining and surface biotinylation in breast cancer cells showed that SPCA2 is partially localized to the plasma membrane where it interacts with the N-terminus of SOC channel Orai1 to elicit the constitutive STIM1-independent Ca^2+^ influx. As a result, the Ca^2+^ dependent nuclear translocation of nuclear factor of activated T cells (NFAT) was upregulated in breast cancer cells. The SPCA2-Orai1 complex thus elicits a novel type of constitutive store-independent Ca^2+^ signalling that promotes breast tumorigenesis.

#### TRP channels in cancer progression

The TRP channels are non-selective cation channels and Ca^2+^ entry pathways in various non-excitable and excitable cells [79]. The superfamily of TRP cation channels are ubiquitously expressed and display an extraordinary diversity of activation mechanisms and functional properties, which enables them to participate in various physiological and pathological conditions, including distinguishing sensations, cell migration and cancer progression [80]. Approximately thirty TRPs have been identified to date and many of them are considered as key players with regard to mechanosensory signalings. Based on sequence homology and channel function, TRP channels can be divided into three main subfamilies: TRPC (Canonical), TRPV (Vanilloid) and TRPM (Melastatin). Many studies have linked specific TRP channels to cancer progression: TRPM1 in human melanoma cells [35,36], TRPV1 in human hepatoblastoma cells [43,44], TRPV2 and TRPM8 in human prostate cancer cells [41,42,45], and TRPC6 in human glioblastoma cells [46,47] (as summarized in Table 1). However, most of these studies are still phenomenological and the downstream Ca^2+^-dependent molecules regulating cell migration and tumor metastasis are often unknown.

#### TRPM7 regulates cell migration though myosin II-based contractility

Among the various TRP cation channels, TRPM7 is the most comprehensively studied class in the context of cell migration. TRPM7 is a bifunctional protein composed of a Ca^2+^- and Mg^2+^- permeable TRP channel fused to a C-terminal α-kinase domain [81], and plays an important role in regulating actomyosin contractility, cell adhesion and directional migration [18,37,82]. TRPM7 can be activated by mechanical force or PLC-activating agonists [18]. Exposure of cells to mechanical stress leads to the opening of the TRPM7 channel, and thereby activates the stretched-activated Ca^2+^ influx at the front of migrating cells [18]. The Ca^2+^ entry through TRPM7 at the front of migrating cells is locally amplified by ER Ca^2+^ release through the IP3R, and thus generates the high Ca^2+^ microdomains (Ca^2+^ flickers) that are required for the guidance of directional movement. In addition, TRPM7 is localized with a Ca^2+^-dependent protease m-calpain at peripheral adhesions, where it regulates focal adhesion assembly and turnover through m-calpain, possibly by mediating the local Ca^2+^ influx near peripheral adhesions [37]. Moreover, a Ca^2+^- and kinase-dependent association between TRPM7 and myosin IIA of the actomyosin cytoskeleton occurs at the proximity of cell adhesions [19]. The α-kinase domain of TRPM7 phosphorylates the myosin IIA heavy chain, and thereby leads to the inhibition of myosin II-based cell contractility and the remodeling of cell adhesions [82]. Taken together, TRPM7-mediated Ca^2+^ flux is an important regulator for directional cell migration through modulating myosin II-based cellular tension and focal adhesion dynamics.

The contributions of TRPM7 to cancer cell migration and tumor metastasis have recently received increasing attention [38-40]. A pro-migratory role of TRPM7 was demonstrated in human nasopharyngeal carcinoma, in which overexpression of TRPM7 protein or increase in its Ca^2+^ channel activity significantly promoted the migration capability, whereas the interference with TRPM7 expression or activation decreased it [40]. It has also been shown that EGF can upregulate the surface expression of TRPM7 proteins and the amplitude of TRPM7 currents, which are important for the basal and the EGF-enhanced cell migration of human lung adenocarcinoma A549 cells [38]. A recent study linking TRPM7 to cell migration and tumor metastasis also suggests the potential of TRPM7 as a strong and independent prognostic marker of poor prognosis of metastatic breast cancer [39]. In breast cancer patients, high levels of TRPM7 mRNA expression were associated with higher incidence of recurrence and metastasis independently of standard clinical parameters. Moreover, TRPM7 expression was functionally required for the invasive migration and the metastasis formation in a mouse xenograft model of human breast cancer [39]. Mechanistic investigations by siRNA-mediated TRPM7 silencing revealed that TRPM7 regulates myosin II-based cytoskeletal contractility and thereby modifies focal adhesion turnover, cell-cell adhesions and polarized cell migration [39]. Therefore, TRPM7 might be part of a mechanosensing complex adopted by cancer cells to drive cancer metastatic phenotypes.

## Conclusions

Remodeling of Ca^2+^ homeostasis is an important event that regulates cancer malignant behaviors. However, there is as yet limited understanding of the role for specific Ca^2+^ signaling in controlling cancer cell migration and tumor metastasis. Future studies could focus on the discovery of potential agents that selectively target cancer cell-specific or tumor vasculature-specific Ca^2+^ influx pathways to facilitate the current diagnostic and therapeutic procedures [72].

## Abbreviations

[Ca2+]i: Intracellular Ca^2+^; ER: Endoplasmic reticulum; EGF: Epidermal growth factor; ECM: Extracellular matrix; GTPase: Guanosine triphosphatase; IP3R: Inositol-1,4,5-trisphosphate receptor; MLC: Myosin light-chain; MLCK: Myosin light chain kinase; NFAT: Nuclear factor of activated T cells; PDGF: Platelet-derived growth factor; Pyk2: Proline-rich tyrosine kinase 2; VEGF: Vascular endothelial growth factor; SAM: Sterile α-motif; STIM: Stromal interaction molecule; SOCE: Store-operated Ca^2+^ entry; TRP: Transient receptor potential

## Competing interests

The authors have no conflicts of interest to declare.

## Authors’ contributions

Y-F C and M-R S collected information, conceived the concept, prepared figures, and drafted the manuscript. Y-T C and W-T C were involved in drafting part of the manuscript. All of the authors read and approved the final manuscript.
